# Impact of malaria control interventions on malaria infection and anaemia in areas with irrigated schemes: a cross-sectional population-based study in Sudan

**DOI:** 10.1186/s12879-021-06929-4

**Published:** 2021-12-14

**Authors:** Khalid Abdelmutalab Elmardi, Ishag Adam, Elfatih Mohammed Malik, Hmooda Toto Kafy, Mogahid Sheikheldien Abdin, Immo Kleinschmidt, Stef Kremers

**Affiliations:** 1 Health Information, Monitoring and Evaluation and Evidence Department, Federal Ministry of Health, Khartoum, Sudan; 2 Department of Health Promotion, Faculty of Health, Medicine and Life Sciences, NUTRIM School of Nutrition and Translational Research in Metabolism, Maastricht, The Netherlands; 3 Department of Obstetrics and Gynecology, Unaizah College of Medicine and Medical Sciences, Qassim University, Unaizah, Saudi Arabia; 4 Faculty of Medicine, University of Khartoum, Khartoum, Sudan; 5 Directorate General of Primary Health Care, Federal Ministry of Health, Khartoum, Sudan; 6 MRC International Statistics and Epidemiology Group, Departments of Infectious Disease Epidemiology, London School of Hygiene and Tropical Medicine, London, UK; 7 Wits Research Institute for Malaria, School of Pathology, Faculty of Health Sciences, University of the Witwatersrand, Johannesburg, South Africa; 8 Southern African Development Community Malaria Elimination Eight Secretariat, Windhoek, Namibia

**Keywords:** Malaria infection, Anaemia, Malaria interventions, Multilevel logistic regression, Malaria indicator survey, Sudan, Irrigated schemes, Low transmission

## Abstract

**Background:**

While the overall burden of malaria is still high, the global technical strategy for malaria advocates for two sets of interventions: vector control-based prevention and diagnosis and prompt effective treatment of malaria cases. This study aimed to assess the performance of malaria interventions on malaria infection and anaemia in irrigated areas in Sudan.

**Methods:**

Based on the Sudan 2016 national malaria indicator survey, data for two states (Gezira and Sennar), characterized by large-irrigated schemes, were analysed. Four community-level malaria interventions were used as contextual variables: utilization of malaria diagnosis, utilization of Artemisinin-based combination therapy (ACT), utilization of long-lasting insecticidal nets (LLINs) and coverage with indoor residual spraying (IRS). Association between these interventions and two outcomes: malaria infection and anaemia, was assessed separately. Malaria infection was assessed in all age groups while anaemia was assessed in children under 5 years. Multilevel multiple logistic regression analysis were conducted.

**Results:**

Among 4478 individuals involved in this study distributed over 47 clusters, the overall malaria infection rate was 3.0% and 56.5% of the children under 5 years (total = 322) were anaemic. Except for IRS coverage (69.6%), the average utilization of interventions was relatively low: 52.3% for utilization of diagnosis, 33.0% for utilization of ACTs and 18.6% for LLINs utilization. The multi-level multiple logistic regression model showed that only IRS coverage was associated with malaria infection (Odds ratio 0.83 per 10% coverage, 95%Confidence Interval (95%CI) 0.74–0.94, p = 0.003) indicating that a higher level of IRS coverage was associated with less malaria infection. Anaemia was not associated with any intervention (all p values larger than 0.1).

**Conclusions:**

Malaria transmission in Gezira and Sennar areas is low. IRS, with insecticide to which vectors are susceptible, is an effective malaria control intervention in irrigated schemes. Community utilization of other interventions was not associated with malaria infection in this study. This may be due to the low utilization of these interventions. However, individual use of LLINs provide personal protection. This study failed to establish an association between anaemia and malaria control interventions in low transmission areas. The higher level of malaria infection in urban areas is a cause for concern.

## Background

Malaria is a major public health problem that is endemic in 87 countries and contributing to around 229 million infections and more than 409 thousand deaths annually [1]. Its level of endemicity and burden depends mainly on the environmental and ecological factors of the area affected [2, 3]. Irrigated schemes provide a suitable environment for mosquito breeding and malaria transmission which needs special consideration in terms of malaria control and intervention selection [4–6]. Additionally, the risk of malaria endemicity is determined by many factors including the area of residence (urban or rural) and the level of interventions coverage [7].

The World Health Organization’s (WHO) updated global technical strategy for malaria calls for universal access to malaria prevention, diagnosis and treatment, recommending two complementarity sets of interventions of (1) vector control-based prevention and (2) timely diagnosis and prompt effective treatment [8]. Currently, the two pertinent vector control interventions are insecticide-treated bed nets (ITNs) and indoor residual spraying (IRS) [8]. The WHO recommends the use of artemisinin-based combination therapy (ACT) for the treatment of malaria cases [9]. For the rational use of the ACTs, all suspected malaria cases should have undergone a parasitological confirmatory diagnostic test [10, 11].

The effectiveness of these malaria interventions has been demonstrated in many studies. The increasing coverage of ITNs, IRS and case management was associated with a decrease in malaria parasite prevalence, anaemia prevalence and all-cause child mortality in Tanzania, Zanzibar, Rwanda, and Malawi [12–15]. Studies showed the effect of ACTs in reducing malaria incidence and transmission [16–19].

The association between malaria and anaemia in endemic countries is well documented [13, 20]. The level of malaria endemicity is a major determinant of the prevalence and severity of malaria-related anaemia [20]. Other causes of anaemia include iron deficiency, nutritional deficiency, drug use, hereditary, chronic diseases, and infections [21–24]. In endemic countries, children under the age of 5 years and pregnant women are the most at-risk population affected by malaria related anaemia [25]. Thus, it is expected that the impact of malaria interventions in controlling malaria would reduce the burden of anaemia [20, 26, 27].

However, coverage and utilization of malaria interventions need to be high enough to demonstrate impact [17].

With the steady decline on malaria during the last two decades, some concerns questioning the effectiveness of the currently adopted malaria interventions came out, especially insecticides in current use [28]. Threats of vectors resistance to insecticide and parasites resistance to Artemisinin and partner drugs, if widely spread in a country, may jeopardize the impact of malaria control [1]. Therefore, and before we get rid of these valuable weapons and/or replace it, there is a need to identify where the challenge is [28] and, of similar importance, to identify how our current tools are performing. Studding how well malaria interventions work in its real-life level of implementation would provide insight for the control programme managers in Sudan and in similar context. This study aimed to assess how the adopted malaria interventions were performing in irrigated areas in Sudan on two outcomes; (1) malaria infection and (2) anaemia.

## Methods

### Study settings

This study was conducted in Sudan which is a low-income African country with an urban population of around 32% [29, 30]^31^]. As a measure of the burden of malaria in country, the parasite prevalence was estimated at 3.3% in the 2012 [32]. The country contributed approximately 1% of the estimated global malaria cases and deaths and approximately 46% of malaria cases to Eastern Mediterranean Region of the WHO [1]. *P. falciparum* is the predominant malaria parasite and *Anopheles arabiensis* is the predominant vector [32, 33]. Malaria transmission in Sudan is mainly affected by seasonal rains, irrigation schemes and urbanization [31]. The two Sudanese states in focus in this study, Gezira and Sennar states, where around 20% of the population are urban, are characterized by large, irrigated schemes covering most of the area where the majority of the population reside. Other climatic conditions and malaria ecology are almost the same in the two states. Because of this, strategies for malaria control in these two states are based on IRS as the predominant vector control intervention together with access to early diagnosis and prompt treatment with ACTs [31]. The use of long-lasting insecticidal nets (LLINs) is not part of the malaria control strategy in these states. However, the use of LLINs was not uncommon among the population of these two states. In 2016, when this study was conducted, the adopted and implemented policy was the use of Artesunate plus Sulfadoxine-Pyrimethamine (AS/SP) and Artemether plus Lumefantrine (AL) as the first- and second-line anti-malaria treatment respectively. By the time of the survey, the LLINs that were in use in Sudan were deltamethrin-impregnated nets (PermaNet2.0 from Vestergaard Frandsen, Lausanne, Switzerland). The insecticide used for IRS in the study areas was bendiocarb (Ficam 80% WP, Bayer company, Leverkusen, Germany; 200 mg of active ingredient per square meter).

### Study design

This study was based on an analysis of the 2016 Sudan malaria indicator survey. Data of Gezira and Sennar states were extracted and analysed. The 2016 Sudan malaria indicator survey is a nationally representative cross-sectional population-based survey conducted in November 2016 just after the rainy season. The survey was designed as a two-stage sampling process. In the first stage, the primary sampling units (clusters) were stratified by rural and urban status, and by state, and randomly selected with probability proportional to population size. In the second stage, 20 households were randomly sampled in each selected cluster. Within each household, all household members were included in the study. Individual and household-related data were collected using a coded pre-tested questionnaire [34] and capillary blood samples were obtained from all eligible individuals. Written informed consent was obtained from participants. Ethical clearance for this study was attained from the Ethical and Technical Review Board at the Directorate of Health Research, Federal Ministry of Health, Sudan.

Obtained data included population socio-demographic characteristics. Collected data also involved whether the household was sprayed with a residual insecticide during the last 6 months, whether the individual slept under a mosquito net the night preceding the survey day and the type of the net in case of yes response, whether an individual who had fever during the 14 days before the survey day was tested parasitologically for malaria; and if positive whether s/he took an ACT for malaria treatment and when. Blood samples were checked in the field for malaria infection and haemoglobin level using malaria rapid diagnostic test (RDT) (SD BIOLINE Malaria Ag Pf/Pv from STANDARD DIAGNOSTICS INC/SD, South Korea) and a field haemoglobin analyser (HemoCue 301 + analyser from Radiometer Group) respectively.

The study aimed to assess the strength of the association between the contextual (community) factors, in terms of malaria control interventions, on individual outcomes (malaria infection and anaemia) while controlling for other contextual and individual variables. Considering the two-stage hierarchical structure of the study and the data arrangement, this study considered two levels for its design with individuals (level 1) nested in their clusters (level 2). As per the multi-level analysis strategy, both clusters and individuals were considered as units of this study.

### Outcome variables

The strength of association of malaria interventions as predictors of the two binary outcomes was assessed separately. Both study outcomes were assessed at an individual level. The first outcome, malaria infection, is defined as the malaria status of study individuals at the time of the survey. It was assessed in all members of the selected households through malaria RDT.

Haemoglobin was measured in all children aged 6–59 months using HemoCue and defined as anaemic if haemoglobin < 110 g/L [35]. Anaemia status was used as the second dichotomous study outcome.

### Contextual independent variables

The four malaria interventions selected as independent variables in this study are (1) utilization of malaria (parasitological) diagnosis, (2) utilization of ACTs, (3) utilization of LLINs and (4) IRS coverage. The first three variables were calculated from the data at the individual level and then aggregated at the cluster level. IRS coverage was similarly calculated at the cluster level from household data. These variables were designed to give an estimation of the population utilization/coverage with malaria interventions at the cluster level. Utilization of malaria diagnosis was defined as the cluster level proportion of individuals with fever who were tested for malaria using malaria parasitological test in the last 14 days prior to the survey date. Similarly, utilization of ACTs was defined as the cluster level proportion of individuals who used ACTs for malaria treatment within 3 days of fever initiation as a response to a parasitologically confirmed malaria in the last 14 days before the survey date. In the same manner, LLINs utilization was defined as the cluster level proportion of individuals who slept under LLIN the night before the survey date. LLINs utilization was calculated irrespective of the availability of the LLINs in the household. IRS coverage was defined as the cluster level proportion of households sprayed with IRS within the last 6 months preceding the survey. These four contextual variables were used in the analysis as continuous variables on a percentage scale. Area of residence, classified into rural and urban, was also used in the analysis to account for its effect.

### Individual-level independent variables

Age in years (as a continuous variable) and sex (dichotomized into male and female) were included in the analysis at the individual level. Individual use of LLINs was also included in the analysis.

### Statistics

The statistical programme used for the analysis was STATA 16.1 (from Stata Corporation LLC, Texas, USA). Since data were collected through a survey and the design was based on multi-stage stratified cluster sampling these features of the study were taken into account in the analysis. Clusters, which was stratified into rural and urban, were specified as the primary sampling unit and households as the secondary sampling unit where all individuals of the household were enrolled. To account for between cluster variation robust (Taylor linearised) standard errors (SE) were estimated. The STATA survey (svy:) commands were used for univariate and bivariate descriptive and inferential statistics. Estimates for subpopulations were done using the STATA subpopulation (subpop) option of the svy prefix.

To describe the overall trend of outcomes and the demographic characteristics of the population, the proportion of the population with the outcome and with the demographic characteristics were calculated as a percentage of the total population who were tested for malaria for the malaria outcome (and as a percentage of the under 5 years children who were tested for anaemia regarding the anaemia outcome). For each malaria intervention, the cluster-level proportion of utilization was estimated and then assigned to every individual in that cluster. Four levels of malaria intervention utilization were defined: low (< 40%), average (40– < 60%), high (60– < 80%) and effective (≥ 80%) utilization. WHO classifies 80% or above as effective community interventions [8].

Taking into account the hierarchical nature and design of the study, an intercept-only multi-level logistic regression analysis (random effects model) was used to assess the strength of the association between malaria infection and the contextual malaria interventions while controlling for the individual (age, sex and individual LLINs use) and other contextual variables (area of residence). All malaria intervention variables as well as other study variables that showed a p-value of less than 0.1 in the bivariate analysis were taken further as potential predictors for malaria infection in the multi-level logistic regression analysis. The cluster was used as the random effect to account for the within-cluster correlation of the outcome (malaria infection). In the analysis, a p-value of less than 0.05 was considered significant.

### Multi-level model building strategy

The strategy for building a multi-level model was based on developing 3 consecutive models (Box 1). The first model was an empty model that only included the cluster (higher level) random effect and it aimed to identify the variation in the outcome that is attributed to cluster variation. Secondly, a model that included malaria interventions was developed to estimate the strength of the association between the variables and the outcome. In the third model, all other study variables were added to the model in order to assess if adding these variables could improve the model.

Box 1: Strategy for multilevel model building

**Table:** 

Models	Model 1: The empty model	Model 2: Malaria interventions model	Model 3: Full model
Variables included	No predictors were included. Only the higher level (cluster) random effect	Malaria interventions (IRS coverage, LLINs utilization and individual use of LLINs). The first two variables are higher-level variables while individual LLINs use is a lower-level variable	All other relevant level-one (age and sex) and level two (area of residence) variables
Objective	To identify the variation in malaria infection that is attributable to the higher level (cluster)	To estimate the strength of the relationship between interventions and malaria infection	To assess if adding these variables could improve the model fit

## Results

### Malaria infection

A total of 4478 surveyed individuals (15.6% of them were under 5 years old) from two irrigated areas in Sudan, distributed over 47 clusters, were included in the analysis of this study. The mean age was 23.3 years (Standard deviation (SD) 19.7, Median 17; Interquartile: first 7 and third 35). Females were representing 57.4% while 80.5% of the surveyed population was living in rural areas.

The overall malaria infection was 3.0% (95%CI 1.7–5.3) with no significant difference (p = 0.199) in infection rate between under 5 years old (2.4%) and 5 years and above (3.1%). *P. falciparum* was the predominant malaria parasite with *P. vivax* representing only 1.5% (2 cases). The mean age of the population with malaria infection was slightly lower (18.7, 95%CI 14.4–23.0 years) than that among those with no infection (23.4, 95%CI 22.4–24.4 years) (p = 0.039). Malaria infection was relatively high among males (3.7%, 95%CI 1.8–7.3) compared to females (2.6%, 95%CI 1.5–4.4) (p = 0.035) while it was relatively low in rural areas (1.8%, 95%CI 1.2–2.8) compared to urban areas (8.1%, 95%CI 3.0–20.2) (p = 0.003) (Table 1).

**Table 1:** Proportion of population with malaria infection and anaemia by sex and area of residence

Variables	Categories	Malaria infection			Anaemia		
		Positive (%, 95%CI) [No.]	Total	*p* value	Anaemic (%, 95%CI) [No.]	Total	*p* value
Sex	Male	(3.7, 1.8–7.3) [70]	1906	0.035	(57.5, 49.2–65.4) [81]	141	0.768
	Female	(2.6, 1.5–4.4) [66]	2572		(55.8, 48.1–63.2) [101]	181	
Area of residence	Rural	(1.8, 1.2–2.8) [65]	3604	0.003	(58.6, 52.4–64.5) [164]	280	0.011
	Urban	(8.1, 3.0–20.2) [71]	874		(42.9, 32.9–53.5) [18]	42	

### Anaemia prevalence

A total of 322 children between 6 months and less than 5 years (representing 7.2% of the total study population) had their haemoglobin level measured, of whom, 56.5% (95%CI 50.8–62.1) were anaemic. Moderate-to-severe anaemia (defined in children under 5 years as a haemoglobin level < 80 g/L [20, 26]) was identified only in 10 (3.1%) children. The mean age of children with anaemia was slightly lower (2.0, 95%CI 1.9–2.2 years) than the mean age of non-anaemic children (2.9, 95%CI 2.6–3.1 years) (p < 0.001). There was no statistically significant difference in anaemia prevalence among children between males (57.5%, 95%CI 49.2–65.4) and females (55.8%, 95%CI 48.1–63.2) (p = 0.768). Childhood anaemia was found relatively more often in urban (58.6%, 95%CI 52.4–64.5) compared to rural communities (42.9%, 95%CI 32.9–53.5) (p < 0.011) (Table 1).

### Utilization of malaria interventions

The average population who utilized malaria interventions ranged from as low as 18.6% for LLINs to as high as 69.6% for IRS. On the other hand, except for the IRS, more than 60% of the population were living in areas of less than 60% malaria interventions’ utilization (low and average utilization of interventions) (Table 2).

**Table 2:** Population distribution by level of utilization of malaria control interventions

Interventions	Level of utilization/coverage of interventions					
	Low (< 40%)	Average (40- < 60%)	High (60- < 80%)	Effective (≥ 80%)	Mean (SD)	Total population
Population utilization of malaria diagnosis [% (No.)]	37.6% (1527)	23.7% (962)	27.1% (1102)	11.6% (472)	52.3% (21.7)	4063
Population utilization of appropriate malaria treatment [% (No.)]	41.1% (988)	35.5% (854)	6.8% (164)	16.6% (398)	33.0% (30.8)	2404
Population utilization of LLINs [% (No.)]	91.6% (3767)	6.5% (266)	1.9% (77)	0 (0%)	18.6% (14.7)	4110
Population coverage with IRS [% (No.)]	2.7% (102)	8.9% (346)	21.5% (826)	66.9% (2573)	69.6% (33.1)	3847

The average cluster-level utilization of interventions is shown in Table 3. Among those with malaria infection, the average cluster-level utilization of LLINs (14.0%, 95%CI 10.8–17.1) varied slightly from the average utilization among those without infection (17.1%, 95%CI 12.9–21.3) (p = 0.070). Similarly, the average cluster-level coverage with IRS varied statistically between those with malaria infection (36.9%, 95%CI 11.4–62.4) compared to those without infection (71.7%, 95%CI 62.9–80.4) (p < 0.001). A large variation was noticed between rural (75.6%, 95%CI 66.5–84.9) and urban (49.7%, 95%CI 24.3–75.1) communities in the average IRS coverage with a borderline p value (p = 0.059).

**Table 3:** Average cluster-level proportion of utilization of malaria interventions by malaria infection, anaemia status and area of residence

Variables	Malaria infection				Anaemia status				Area of residence			
	Categories	Mean (95%CI)	Total	*p* value	Categories	Mean (95%CI)	Total	*p* value	Categories	Mean (95%CI)	Total	*p* value
Population utilization of malaria diagnosis (%)	Positive	54.1 (46.5–61.6)	126	0.192	Anaemic	46.7 (38.8–54.6)	168	0.489	Rural	48.4 (40.9–55.9)	3339	0.552
	Negative	49.2 (42.6–55.8)	4000		Non-anaemic	49.0 (40.0–58.0)	129		Urban	53.4 (38.4–68.3)	787	
Population utilization of appropriate malaria treatment (%)	Positive	33.3 (28.7–38.0)	122	0.965	Anaemic	32.9 (19.9–45.9)	148	0.438	Rural	31.1 (19.9–42.3)	3002	0.242
	Negative	33.2 (23.5–42.8)	3518		Non-anaemic	29.6 (20.3–39.0)	122		Urban	42.8 (26.5–59.0)	638	
Population utilization of LLINs (%)	Positive	14.0 (10.8–17.1)	136	0.070	Anaemic	20.1 (13.3–26.9)	140	0.352	Rural	18.1 (13.0–23.2)	3236	0.203
	Negative	17.1 (12.9–21.3)	4342		Non-anaemic	18.4 (13.1–23.7)	182		Urban	13.0 (6.7–19.2)	874	
Population coverage with IRS (%)	Positive	36.9 (11.4–62.4)	135	< 0.001	Anaemic	69.4 (58.0–80.9)	163	0.827	Rural	75.6 (66.5–84.9)	3604	0.059
	Negative	71.7 (62.9–80.4)	3975		Non-anaemic	70.1 (58.3–82.0)	135		Urban	49.7 (24.3–75.1)	874	

The overall individual use of LLINs was 15.7% which was not statistically different among rural (16.4%, 95%CI 12.3–21.7) compared to urban (12.6%, 95%CI 7.7–20.0) population (p = 0.343). Weak statistical evidence was seen in individual use of LLINs among people with malaria infection (15.9%, 95%CI 12.3–20.4) compared to those without infection (8.8%, 95%CI 3.8–19.4) (p = 0.134). The difference in LLINs individual use was not significant among anaemic (22.2%, 95%CI 14.6–32.4) compared to non-anaemic (29.7%, 95%CI 19.8–42.0) children (p = 0.139).

When considering anaemia, the study identified no significant statistical differences in the averages of cluster-level utilization of malaria interventions among anaemic compared to non-anaemic children (all p values were far larger than 0.1) as shown in Table 3.

### Association between malaria infection and malaria interventions

Table 4 shows findings of the multilevel models built to estimate the association between malaria infection and malaria interventions. The null model demonstrates a cluster variance component of 1.89 (95%CI 0.98–3.63) and an intra-class correlation coefficient (ICC) of 0.37 (95%CI 0.23–0.52) indicating that 37% of the variation in malaria infection is due to differences in clusters’ characteristics rather than individual-level characteristics. The likelihood ratio test was statistically significant (p = 0.008) reflecting that model 3 is an improvement of model 2 and hence considered as the final model. The final multi-level multiple logistic regression model (model 3) showed that IRS coverage is significantly associated with malaria infection where the odds of malaria infection is decreasing by 17% for every 10% increase in IRS coverage (adjusted Odds ratio (aOR) 0.83 per 10% coverage, 95%CI 0.73–0.94, p = 0.002). While LLINs utilization was not found to be significantly associated with the malaria infection (aOR 0.91, 95%CI 0.69–1.22, p = 0.541), individual LLINs use demonstrated a protective effect on malaria infection but with borderline strength of evidence (aOR 0.54, 95%CI 0.28–1.04, p = 0.064).

**Table 4:** Association between malaria infection and malaria interventions

Models		Multi-level logistic regression models		
	Multi-level simple logistic regression	Model 1: The empty model	Model 2: Malaria interventions model	Model 3: Full model
	cOR (95%CI)	aOR (95%CI)	aOR (95%CI)	aOR (95%CI)
Fixed part				
IRS coverage (per 10% coverage)	0.85 (0.76–0.96) p = 0.008	–	0.81 (0.72–0.91) p < 0.001	0.83 (0.73–0.94) p = 0.002
LLINs utilization (per 10% utilization)	0.88 (0.63–1.22) p = 0.441	–	0.90 (0.67–1.20) p = 0.480	0.91 (0.69–1.22) p = 0.541
Timely access to malaria diagnosis (per 10% utilization)	0.93 (0.74–1.17) p = 0.540	–	–	–
Timely access to appropriate malaria treatment (per 10% utilization)	1.01 (0.84–1.21) p = 0.936	–	–	–
Individual use of LLINs				
No	1	–	1	1
Yes	0.53 (0.28–1.02) p = 0.056	–	0.53 (0.28–1.02) p = 0.056	0.54 (0.28–1.04) p = 0.064
Area of residence			–	
Rural (Reference)	1	–	–	1
Urban	2.02 (1.04–4.65) p = 0.038	–	–	1.48 (0.71–3.08) p = 0.291
Sex				
Male (Reference)	1	–	–	1
Female	0.72 (0.50–1.03) p = 0.075	–	–	0.73 (0.51–1.06) p = 0.101
Age, per year	0.99 (0.98–1.00) p = 0.019	–	–	0.98 (0.97–1.00) p = 0.007
Random part				
Variance component (cluster)	–	1.89 (95%CI 0.98–3.63) ICC = 0.37 (95%CI 0.23–0.52)	–	–

Estimates of two-level logistic regression models for the association between malaria infection and malaria interventions in areas with irrigated schemes, Sudan 2016

*cOR* crude odds ratio, *95%CI* 95% confidence interval, *aOR* adjusted odds ratio, *OR* odds ratio, *ICC* intraclass correlation coefficient

### Association between anaemia and malaria interventions

Since the bivariate analysis demonstrated no statistically significant association between malaria interventions and anaemia in children (Table 5), no further multiple regression analysis was considered for anaemia.

**Table 5:** Association between anaemia and malaria interventions

Model	Multi-level simple logistic regression cOR (95%CI)
Fixed part	
IRS coverage (per 10% coverage)	0.99 (0.93–1.06) p = 0.851
LLINs utilization (per 10% utilization)	1.06 (0.93–1.22) p = 0.374
Timely access to malaria diagnosis (per 10% utilization)	0.96 (0.87–1.06) p = 0.418
Timely access to appropriate malaria treatment (per 10% utilization)	1.03 (0.96–1.12) p = 0.394
Individual use of LLINs	
No	1
Yes	0.68 (0.40–1.13) p = 0.137

Estimates of two-level simple logistic regression for the association between anaemia and malaria interventions in areas with irrigated schemes, Sudan 2016

*cOR* crude odds ratio, *95%CI* 95% confidence interval

## Discussion

This study showed that malaria infection in the Sudanese irrigated areas of Gezira and Sennar states was low. The level of infection is in line with classifying the area as a low malaria transmission setting as previously described by Noor *et.al.* [31]. This is further supported by the mean age of the population with malaria infection in this study (18.7 years) which is higher than the mean age of the infected population in moderate to high malaria transmission settings where the burden is concentrated in young children [36, 37]. A previous study from these areas showed a reduction in malaria prevalence to below 2% over 10 years (1999–2009) [38]. It is not clear if this change in prevalence was due to intense malaria control activities or due to other factors. However, a decreasing trend in malaria infection rate in Sudan was noticed with the increasing coverage of malaria control intervention in recent years [39] and previous peaks of infection were associated with interruption of IRS intervention in these areas [38].

The utilization of malaria interventions in Gezira and Sennar was found to be relatively low. The majority of the population was living in areas where utilization of malaria diagnosis, effective malaria treatment and LLINs were far below the targeted community effectiveness level of ≥ 80% [8]. IRS was an exception, and the vast majority of the population in this study was living in areas with high and effective IRS coverage. Globally, access to and utilization of malaria control interventions are challenged with the low levels of coverage [1, 40, 41]. Such a situation may reduce the expected community benefit of these interventions to eliminate malaria from endemic areas. Nonetheless, increasing coverage of one or more interventions has been shown to be associated with a reduction in malaria morbidity and mortality in many studies [13, 41–44].

Among the assessed malaria control interventions in this study, only IRS coverage was found to be a predictor of protection against malaria infection after controlling for age, sex and area of residence. Other tested malaria interventions showed no statistically significant association with malaria infection as a community protective measure. This could be, in part, due to the low utilization level of these interventions [41]. Low utilization of LLINs is to be expected since it was not a targeted intervention in these areas. This level of LLINs coverage and utilization is probably due to individual interest and/or residual from previous distribution campaigns related to previous strategy and study projects [45]. However, individual use of LLINs in this study showed some protection against malaria infection. This effect could be due to the effect of nets as a mechanical barrier against mosquitoes in addition to the role of the insecticide. Nevertheless, vector resistance to deltamethrin, the insecticide used for the treatment of LLINs that were available in Sudan by the time of the study, was documented in these areas [33, 46]. While we could not rule out the effect of insecticide in these nets, it is not clear if the lower effect of community utilization of LLINs on malaria infection was due to the lower utilization or the insecticide resistance [47]. Reasons for low utilization of malaria diagnosis and ACTs need to be identified and addressed. This study did not elaborate on whether the low utilization of diagnosis and ACTs was due to gaps in the health system (i.e. access to health facilities, service availability, cost, providers failing to test for malaria, … etc.) or due to population behaviour (i.e. seeking timely care at the onset of symptoms). Concurrently and during the period preceding this study, decreasing efficacy of AS/SP in Sudan was reported which was mainly due to the failing SP component [48]. On the other hand, this study showed that higher coverage with IRS is associated with decreasing odds, and hence, a lower risk of malaria infection. The effectiveness of IRS as a malaria control intervention [49–54] is dependent on the susceptibility of the vector to the insecticide in use, the diligence of its application as well as the wall surface material [55, 56]. Residual efficacy of IRS with bendiocarb was found to range between 2 and 7 months [55–57]. In Gezira and Sennar, two rounds of IRS were implemented as a routine in most of the years. Training and supervision of IRS implementation were regular exercises of each round. Bendiocarb-based IRS was in use in these areas since 2012 as a result of increasing insecticide resistance to previously used pyrethroids. Recent studies showed that carbamate is an effective IRS insecticide in these areas [33, 45]. With the findings from this study, it is evident that carbamate-based IRS is still effective in reducing malaria infection and hence the disease burden as shown elsewhere [53]. Nonetheless and due to the operational difficulties of IRS application in rooms with modern furniture, parts of the urban areas in Gezira state were routinely excluded from spraying by the malaria control programme. This may have resulted in the difference in IRS coverage between rural and urban areas detected by this study and may explain the higher prevalence of malaria infection in urban compared to rural areas. In the final model, after adjusting for IRS coverage, area of residence was not significantly associated with malaria infection probably due to the association between IRS coverage and rural versus urban area.

The current study additionally assessed the relationship between malaria interventions and childhood anaemia. More than half of the children under the age of five in this study were anaemic. However, this study failed to establish an association between malaria interventions and anaemia in under 5 children in contrast to many other studies [12–15]. It is not clear why such association was not present in this study. This may be explained by the low malaria infection rate in our study and because the burden of infection was not concentrated in young children. In such a low level of malaria transmission chronic malaria and repeated malaria infections are less common and therefore, may not be a major cause of anaemia. Studies from low to moderate transmission settings, including from Sudan [34], showed that anaemia was associated with malaria infection but not with the level of malaria transmission [27, 58–60]. Moderate-to-severe anaemia (haemoglobin < 80 g/L) is a more sensitive measure to the association between anaemia and malaria compared to anaemia (haemoglobin < 110 g/L), which was used in this study [20, 26]. However, the very low prevalence of moderate-to-severe anaemia identified in this study is further supporting the absence of such association in low malaria transmission areas [20, 61].

This study has strengths and limitations. Its modelling was based on a multi-level regression analysis. Hierarchical analysis, which was used here, gives such studies more precision than the regular regression models since multi-level analysis accounts for within-cluster correlation. The study highlighted the relationship between malaria interventions and malaria infection and anaemia in irrigated areas in low malaria transmission settings. It examined how these interventions were performing in real life utilization levels with varying degrees of intervention coverage. Limitations of this study include tools and methods of assessing outcomes. Malaria infection was assessed in this study using RDTs which has its own shortcomings in malaria diagnosis. All RDTs have certain lower-level limits of parasitaemia below which they may give false negative results, a common condition in low transmission settings. Combo RDT tests (which detects two or more parasite species) are generally less sensitive to one or both species. However, RDTs are operationally more reliable in surveys, due to technical and practical problems of assuring the quality of malaria microscopy. Moreover, parasites that not expressing histidine-rich protein (HRP) may not be detected by HRP-based RDTs. On the other hand, the lower number of children tested for anaemia in this study may reduce the power of detecting the relationship between interventions and anaemia. One more limitation of this study is the assessment of the LLINs use which was based on whether individuals slept under LLIN the nigh prior to the survey as routinely measured by malaria indicator surveys. This is an estimation of use but is not an accurate representation of long-term use. Another limitation is that this study did not address the larval source management activities (LSM) which are partially implemented in some urban areas of the study. This was due to difficulties in identifying areas (clusters) where LSM was implemented as well as the operational challenges in defining and measuring the level of population coverage with LSM. However, the findings of this research have provided good insight into the topic and raised some unanswered questions for future research including what the reasons for low utilization of interventions are; supply or demand.

## Conclusions

The malaria infection rate in irrigated areas of Gezira and Sennar states in Sudan was low. The utilization of malaria interventions is generally below the community effectiveness level except for the IRS. IRS was found to protect against malaria infection. In urban areas, where high coverage of IRS may be more difficult to achieve, malaria infection prevalence was higher than in rural areas. The community-level low utilization of malaria diagnosis and ACTs as well as the low utilization of LLINs may be the most important explanation of the absence of the relation with malaria infection. However, with this low level of community utilization there was some evidence that the individual use of LLINs was providing personal protection against malaria infection. This study was unable to establish a relationship between anaemia and malaria interventions in low malaria transmission areas of the current study. It is recommended that vector control with IRS, should be intensified due to its documented effectiveness in this study. In areas where high levels of IRS coverage are difficult to maintain, it may be necessary to intensify alternative means of protection against malaria, such as LSM, or LLIN distributions. The susceptibility of vectors to the insecticides used should be continuously monitored to ensure the sustained efficacy of this intervention.

## Data Availability

The original survey data of this research is available from the directorate of Communicable and Non-communicable diseases Control, Federal Ministry of Health, Sudan. Request for data could be addressed to the director of this directorate, if needed.

## Abbreviations

ACT: Artemisinin-based combination therapy
AL: Artemether plus Lumefantrine
AS/SP: Artesunate plus Sulfadoxine-Pyrimethamine
IRS: Indoor residual spraying
ITNs: Insecticide-treated bed nets
LLINs: Long-lasting insecticidal nets
LSM: Larval source management activities
OR: Odds ratio
*P.** falciparum*: *Plasmodium falciparum*
*P.** vivax*: *Plasmodium vivax*
RDT: Malaria rapid diagnostic test
WHO: World Health Organization
95%CI: 95% Confidence interval

## References

[1] World Health Organization. World Malaria Report 2020: 20 years of global progress and challenges. Geneva; 2020.

[2] Bannister-Tyrrell M, Verdonck K, Hausmann-Muela S, Gryseels C, Muela Ribera J, Peeters GK (2017). Defining micro-epidemiology for malaria elimination: systematic review and meta-analysis. Malar J.

[3] Giardina F, Kasasa S, Sié A, Utzinger J, Tanner M, Vounatsou P (2014). Effects of vector-control interventions on changes in risk of malaria parasitaemia in sub-Saharan Africa: a spatial and temporal analysis. Lancet Glob Health.

[4] Lee E, Burkhart J, Olson S, Billings AA, Patz JA, Harner EJ (2016). Relationships of climate and irrigation factors with malaria parasite incidences in two climatically dissimilar regions in India. J Arid Environ.

[5] Kibret S, Wilson GG, Tekie H, Petros B (2014). Increased malaria transmission around irrigation schemes in Ethiopia and the potential of canal water management for malaria vector control. Malar J.

[6] De Plaen R, Seka M-L, Koutoua A (2004). The paddy, the vector and the caregiver: lessons from an ecosystem approach to irrigation and malaria in Northern Côte d’Ivoire. Acta Trop.

[7] WHO. WHO malaria terminology Global Malaria Programme. Geneva: World Health Organization; 2019.

[8] WHO. Global technical strategy for malaria 2016–2030. 2021 updat. Geneva; 2021.

[9] WHO. Antimalarial drug combination therapy report of a WHO Technical Consultation. Geneva; 2001.

[10] WHO. WHO Guidelines for malaria. Geneva: World Health Organization; 2021.

[11] WHO. Guidelines for the treatment of malaria. Geneva: World Health Organization; 2015. 26020088

[12] Hershey CL, Florey LS, Ali D, Bennett A, Luhanga M, Mathanga DP (2017). Malaria control interventions contributed to declines in malaria parasitemia, severe anemia, and all-cause mortality in children less than 5 years of age in Malawi, 2000–2010. Am J Trop Med Hyg.

[13] Eckert E, Florey LS, Tongren JE, Salgado SR, Rukundo A, Habimana JP (2017). Impact evaluation of malaria control interventions on morbidity and all-cause child mortality in Rwanda, 2000–2010. Am J Trop Med Hyg.

[14] Aregawi MW, Ali AS, Al-mafazy A, Molteni F, Katikiti S, Warsame M (2011). Reductions in malaria and anaemia case and death burden at hospitals following scale-up of malaria control in Zanzibar, 1999–2008. Malar J.

[15] Smithson P, Florey L, Salgado SR, Hershey CL, Masanja H, Bhattarai A (2015). Impact of malaria control on mortality and anemia among Tanzanian children less than five years of age, 1999–2010. PLoS ONE.

[16] Gimba B, Bala SI (2017). Modeling the impact of bed-net use and treatment on malaria transmission dynamics. Int Scholar Res Notices.

[17] Mbah MLN, Parikh S, Galvani AP (2015). Comparing the impact of artemisinin-based combination therapies on malaria transmission in Sub-Saharan Africa. Am J Trop Med Hyg.

[18] Sagara I, Fofana B, Gaudart J, Sidibe B, Togo A, Toure S (2012). Repeated artemisinin-based combination therapies in a malaria hyperendemic area of Mali: efficacy, safety, and public health impact. Am J Trop Med Hyg.

[19] Bhattarai A, Ali AS, Kachur SP, Mårtensson A, Abbas AK, Khatib R (2007). Impact of artemisinin-based combination therapy and insecticide-treated nets on malaria burden in Zanzibar. PLoS Med.

[20] White NJ (2018). Anaemia and malaria. Malar J.

[21] WHO. The Global Prevalence of Anaemia in 2011. Geneva: World Health Organization; 2015.

[22] Jin L, Yeung LF, Cogswell ME, Ye R, Berry RJ, Liu J (2010). Prevalence of anaemia among pregnant women in south-east China, 1993–2005. Public Health Nutr.

[23] Janus J, Moerschel SK (2010). Evaluation of anemia in children. Am Fam Phys.

[24] Bates I, McKew S, Sarkinfada F (2007). Anaemia: a useful indicator of neglected disease burden and control. PLoS Med.

[25] Juan Q, Machado S, Alberto T, Silvia B, Alberto M, Myriam A-H (2011). Malaria-related anaemia: a Latin American perspective. Mem Inst Oswaldo Cruz.

[26] Mathanga DP, Campbell CH, vanden Eng J, Wolkon A, Bronzan RN, Malenga GJ (2010). Comparison of anaemia and parasitaemia as indicators of malaria control in household and EPI-health facility surveys in Malawi. Malar J.

[27] Castelli F, Sulis G, Caligaris S (2014). The relationship between anaemia and malaria: apparently simple, yet controversial. Trans R Soc Trop Med Hyg.

[28] Lindsay SW, Thomas MB, Kleinschmidt I (2021). Threats to the effectiveness of insecticide-treated bednets for malaria control: thinking beyond insecticide resistance. Lancet Glob Health.

[29] Central Bureau of Statistics. Sudan Population Data Sheet 2018. Khartoum, Sudan; 2018.

[30] Federal Ministry of Health. National Health Sector Strategic Plan (2012–2016). Khartoum, Sudan: Federal Ministry of Health; 2011.

[31] Noor AM, ElMardi KA, Abdelgader TM, Patil AP, Amine AAA, Bakhiet S (2012). Malaria risk mapping for control in the Republic of Sudan. Am J Trop Med Hyg.

[32] Federal Ministry of Health. Malaria Indicator Survey 2012 in the Republic of Sudan. Khartoum, Sudan; 2013.

[33] Ismail BA, Kafy HT, Sulieman JE, Subramaniam K, Thomas B, Mnzava A (2018). Temporal and spatial trends in insecticide resistance in Anopheles arabiensis in Sudan: outcomes from an evaluation of implications of insecticide resistance for malaria vector control. Parasit Vectors.

[34] Elmardi KA, Adam I, Malik EM, Ibrahim AA, Elhassan AH, Kafy HT (2020). Anaemia prevalence and determinants in under 5 years children: findings of a cross-sectional population-based study in Sudan. BMC Pediatr.

[35] WHO. Haemoglobin concentrations for the diagnosis of anaemia and assessment of severity. Geneva, Switzerland; 2011.

[36] Vestergaard LS, Lusingu JP, Nielsen MA, Mmbando BP, Dodoo D, Akanmori BD (2008). Differences in human antibody reactivity to *Plasmodium falciparum *variant surface antigens are dependent on age and malaria transmission intensity in Northeastern Tanzania. Infect Immun.

[37] Lusingu JPA, Vestergaard LS, Mmbando BP, Drakeley CJ, Jones C, Akida J (2004). Malaria morbidity and immunity among residents of villages with different *Plasmodium falciparum* transmission intensity in North-Eastern Tanzania. Malar J.

[38] Mirghani SE, Nour BY, Bushra SM, El HI, Snow RW, Noor AM (2010). The spatial-temporal clustering of *Plasmodium falciparum* infection over eleven years in Gezira State. Sudan Malar J.

[39] National Malaria Control Progarmme. Malaria Programme Review 2001–2012. Khartoum, Sudan; 2013.

[40] Runge M, Snow RW, Molteni F, Thawer S, Mohamed A, Mandike R (2020). Simulating the council-specific impact of anti-malaria interventions: a tool to support malaria strategic planning in Tanzania. PLoS ONE.

[41] Griffin JT, Hollingsworth TD, Okell LC, Churcher TS, White M, Hinsley W (2010). Reducing *Plasmodium falciparum* malaria transmission in Africa: a model-based evaluation of intervention strategies. PLoS Med.

[42] Thwing J, Eckert E, Dione DA, Tine R, Faye A, Yé Y (2017). Declines in malaria burden and all-cause child mortality following increases in control interventions in Senegal, 2005–2010. Am J Trop Med Hyg.

[43] Karema C, Aregawi MW, Rukundo A, Kabayiza A, Mulindahabi M, Fall IS (2012). Trends in malaria cases, hospital admissions and deaths following scale-up of anti-malarial interventions, 2000–2010, Rwanda. Malar J.

[44] Steketee RW, Campbell CC (2010). Impact of national malaria control scale-up programmes in Africa: magnitude and attribution of effects. Malar J.

[45] Kafy HT, Ismail BA, Mnzava AP, Lines J, Abdin MSE, Eltaher JS (2017). Impact of insecticide resistance in *Anopheles arabiensis* on malaria incidence and prevalence in Sudan and the costs of mitigation. Proc Natl Acad Sci.

[46] Implications of Insecticide Resistance Consortium (2018). Implications of insecticide resistance for malaria vector control with long-lasting insecticidal nets: trends in pyrethroid resistance during a WHO-coordinated multi-country prospective study. Parasit Vectors.

[47] Stebbins RC, Emch M, Meshnick SR (2018). The effectiveness of community bed net use on malaria parasitemia among children less than 5 years old in Liberia. Am J Trop Med Hyg.

[48] Adeel AA, Elnour FAA, Elmardi KA, Abd-Elmajid MB, Elhelo MM, Ali MS (2016). High efficacy of artemether-lumefantrine and declining efficacy of artesunate + sulfadoxine-pyrimethamine against *Plasmodium falciparum* in Sudan (2010–2015): evidence from in vivo and molecular marker studies. Malar J.

[49] Hien AS, Soma DD, Sawadogo SP, Poda SB, Namountougou M, Ouédraogo GA (2020). Effect of Bendiocarb (Ficam® 80% WP) on entomological indices of malaria transmission by indoor residual spraying in Burkina Faso, West Africa. Adv Entomol.

[50] Corrêa APSA, Galardo AKR, Lima LA, Câmara DCP, Müller JN, Barroso JFS (2019). Efficacy of insecticides used in indoor residual spraying for malaria control: an experimental trial on various surfaces in a “test house”. Malar J.

[51] Musiime AK, Smith DL, Kilama M, Rek J, Arinaitwe E, Nankabirwa JI (2019). Impact of vector control interventions on malaria transmission intensity, outdoor vector biting rates and Anopheles mosquito species composition in Tororo, Uganda. Malar J.

[52] Gimnig JE, Otieno P, Were V, Marwanga D, Abong’o D, Wiegand R (2016). The effect of indoor residual spraying on the prevalence of malaria parasite infection, clinical malaria and anemia in an area of perennial transmission and moderate coverage of insecticide treated nets in Western Kenya. PLoS ONE.

[53] Katureebe A, Zinszer K, Arinaitwe E, Rek J, Kakande E, Charland K (2016). Measures of malaria burden after long-lasting insecticidal net distribution and indoor residual spraying at three sites in Uganda: a prospective observational study. PLOS Med.

[54] Pluess B, Tanser FC, Lengeler C, Sharp BL (2010). Indoor residual spraying for preventing malaria. Cochrane Database Syst Rev.

[55] Lo C, Dia AK, Dia I, Niang EHA, Konaté L, Faye O (2019). Evaluation of the residual efficacy of indoor residual spraying with bendiocarb (FICAM WP 80) in six health districts in Senegal. Malar J.

[56] Dengela D, Seyoum A, Lucas B, Johns B, George K, Belemvire A (2018). Multi-country assessment of residual bio-efficacy of insecticides used for indoor residual spraying in malaria control on different surface types: results from program monitoring in 17 PMI/USAID-supported IRS countries. Parasit Vectors.

[57] Djènontin A, Aïmihouè O, Sèzonlin M, Damien GB, Ossè R, Soukou B (2013). The residual life of bendiocarb on different substrates under laboratory and field conditions in Benin, Western Africa. BMC Res Notes.

[58] Ntenda PAM, Chilumpha S, Mwenyenkulu ET, Kazambwe JF, El-Meidany W (2019). Clinical malaria and the potential risk of anaemia among preschool-aged children: a population-based study of the 2015–2016 Malawi micronutrient survey. Infect Dis Poverty.

[59] Mensah-Brown HE, Abugri J, Asante KP, Dwomoh D, Dosoo D, Atuguba F (2017). Assessing the impact of differences in malaria transmission intensity on clinical and haematological indices in children with malaria. Malar J.

[60] Kilama M, Lindsay SW, Greenhouse B, Arinaitwe E, Kamya MR, Staedke SG (2015). Malaria transmission, infection, and disease at three sites with varied transmission intensity in Uganda: implications for malaria control. Am J Trop Med Hyg.

[61] Korenromp EL, Armstrong-Schellenberg JRMM, Williams BG, Nahlen BL, Snow RW (2004). Impact of malaria control on childhood anaemia in Africa—a quantitative review. Trop Med Int Health.

